# High Levels of NfL, GFAP, TAU, and UCH-L1 as Potential Predictor Biomarkers of Severity and Lethality in Acute COVID-19

**DOI:** 10.1007/s12035-023-03803-z

**Published:** 2023-11-24

**Authors:** Andreza Lemos Salvio, Renan Amphilophio Fernandes, Helena França Alcaraz Ferreira, Larissa Araujo Duarte, Elisa Gouvea Gutman, Jessica Vasques Raposo-Vedovi, Carlos Henrique Ferreira Ramos Filho, Wagner Luis da Costa Nunes Pimentel Coelho, Giselle Fazzioni Passos, Maria Emília Cosenza Andraus, João Paulo da Costa Gonçalves, Marta Guimarães Cavalcanti, Marisa Pimentel Amaro, Rafael Kader, Roberto de Andrade Medronho, Cláudia Pinto Figueiredo, Luciane Almeida Amado-Leon, Soniza Vieira Alves-Leon

**Affiliations:** 1 Laboratory of Translacional Neurosciences, Biomedical Institute, Federal University of the State of Rio de Janeiro-UNIRIO, Rio de Janeiro, 22290-240 Brazil; 2 Clementino Fraga Filho University Hospital, Federal University of Rio de Janeiro, Rio de Janeiro, 21941-901 Brazil; 3 Laboratory of Technological Development in Virology, Oswaldo Cruz Institute/Fiocruz, Rio de Janeiro, 21040-900 Brazil; 4 School of Pharmacy, Federal University of Rio de Janeiro, Rio de Janeiro, 21941-902 Brazil; 5 Epidemiology and Evaluation Service, Clementino Fraga Filho University Hospital, Federal University of Rio de Janeiro, Rio de Janeiro, 21941-901 Brazil; 6 School of Medicine, Post-Graduate Program in Infectious and Parasitic Diseases, Federal University of Rio de Janeiro, Rio de Janeiro, 21941-901 Brazil

**Keywords:** Precision medicine, COVID-19, Neurofilament light chain, TAU protein, Glial Fibrillary Acidic Protein, Ubiquitin Carboxy-terminal Hydrolase L1

## Abstract

Few studies showed that neurofilament light chain (NfL), glial fibrillary acidic protein (GFAP), total tubulin-associated unit (TAU), and ubiquitin carboxy-terminal hydrolase-L1 (UCH-L1) may be related to neurological manifestations and severity during and after SARS-CoV-2 infection. The objective of this work was to investigate the relationship among nervous system biomarkers (NfL, TAU, GFAP, and UCH-L1), biochemical parameters, and viral loads with heterogeneous outcomes in a cohort of severe COVID-19 patients admitted in Intensive Care Unit (ICU) of a university hospital. For that, 108 subjects were recruited within the first 5 days at ICU. In parallel, 16 mild COVID-19 patients were enrolled. Severe COVID-19 group was divided between “deceased” and “survivor.” All subjects were positive for SARS-CoV-2 detection. NfL, total TAU, GFAP, and UCH-L1 quantification in plasma was performed using SIMOA SR-X platform. Of 108 severe patients, 36 (33.33%) presented neurological manifestation and 41 (37.96%) died. All four biomarkers — GFAP, NfL, TAU, and UCH-L1 — were significantly higher among deceased patients in comparison to survivors (*p* < 0.05). Analyzing biochemical biomarkers, higher Peak Serum Ferritin, D-Dimer Peak, Gamma-glutamyltransferase, and C-Reactive Protein levels were related to death (*p* < 0.0001). In multivariate analysis, GFAP, NfL, TAU, UCH-L1, and Peak Serum Ferritin levels were correlated to death. Regarding SARS-CoV-2 viral load, no statistical difference was observed for any group. Thus, Ferritin, NFL, GFAP, TAU, and UCH-L1 are early biomarkers of severity and lethality of SARS-COV-2 infection and may be important tools for therapeutic decision-making in the acute phase of disease.

**Supplementary Information:**

The online version contains supplementary material available at 10.1007/s12035-023-03803-z.

## Background

Severe Acute Respiratory Syndrome Coronavirus 2 (SARS-CoV-2) is the etiological agent of Coronavirus disease 2019 (COVID-19) and can be responsible for a wide range of different outcomes, varying from asymptomatic infection, mild disease, and even severe acute respiratory syndrome (SARS) and death [1, 2]. Although the end of the Public Health Emergency of International Concern for COVID-19 was decreed by the World Health Organization (WHO), on May 5, 2023 [3], it brought us all relevant questioning, mainly regarding the patients’ management and monitoring. Since the COVID-19 outbreak, there is an intense and constant search for understanding the host’s response to infection and its relation to different outcomes. Several factors may be related to good or poor COVID-19 prognosis. Important marks remain in our society, and it reinforces how relevant it is to know and predict poor prognosis to act fast enough and take the most reasonable decisions and therapeutic approaches [3]. Demographic features, viral load, host genetic susceptibility, blood biomarkers, and innate immunologic and inflammatory response, for example, may be related to heterogenous COVID-19 outcomes [4, 5]. SARS-CoV-2 viral loads may be an important factor which is not clear yet if it is directly related to stronger inflammatory response and disease severity or not [6, 7]. Also, serum biomarkers may also be important tools for predicting worse outcome; for example, serum Ferritin levels (peak) have been associated to longer severe disease and death [8–10]. Therefore, it is important to consider such factors combined to have a clearer picture of both the whole scenario and each individual infection course, mostly when it comes to poor prognosis and COVID-19 severe and fatal cases. It is essential to apply and investigate the most adequate tools in precision medicine to better assess each patient predictive factors of severity.

When it comes to the neurological manifestations and survival rates, some neurological biomarkers may have a huge impact as predictive factors of the patient’s outcome, but still more efforts are necessary so they can be applied on daily monitoring of acute cases. Studies have shown a relationship between neurological biomarkers, such as neurofilament light chain (NfL), glial fibrillary acidic protein (GFAP), total tubulin-associated unit (TAU) protein, and ubiquitin carboxy-terminal hydrolase L1 (UCH-L1), with acute and long-term neurological symptom manifestations induced by SARS-CoV-2. Also, they can act as potential tools for severity and mortality assessment during the acute infection [11–13]. NfL — a cytoskeletal intermediate filament protein of neurons — consists of an important biomarker of axonal damage and degeneration, strongly related to several neurological diseases, such as Multiple Sclerosis and Acute Disseminated Encephalomyelitis [14]. UCH-L1 — a neuron specific cytoplasmic deubiquitinating enzyme — is an important neuronal cell body injury and apoptosis biomarker, while GFAP is a structural protein of astrocytes, which is released in higher levels during brain injury and degeneration. Furthermore, both UCH-L1 and GFAP levels can be related to brain injury [15]. Tau protein acts in the stabilization of microtubules of neurons and axonal transportation. Its levels can be higher in taupathies, such as Alzheimer’s disease, and can be important markers of neuron damage or neurodegeneration process [16, 17].

These biomarkers are very well established for neurological disorder investigation using cerebrospinal fluid (CSF) samples, but lately, their levels in peripheral blood can be accurately quantified using ultrasensitive techniques, such as Single Molecule Array (SiMoA) [4, 12]. Therefore, it is important to understand and clarify the relation among these neurological biomarkers and COVID-19 outcomes in order to strategically apply them as monitoring tools not only for Intensive Care Unit (ICU) but also for early admission of COVID-19 patients.

Thus, the aim of this study was to investigate the relationship among nervous system biomarkers (NfL, TAU, GFAP, and UCH-L1), biochemical parameters, viral loads, and heterogeneous outcomes in a cohort of severe COVID-19 patients at admission in the ICU of a public hospital.

## Methods

### Study Population

This study recruited and included subjects between July 2020 and October 2021. A total of 706 patients were admitted in the ICU of the Clementino Fraga Filho University Hospital, Federal University of Rio de Janeiro (HUCFF/UFRJ) in Rio de Janeiro, RJ, Brazil. From this population, we enrolled a representative sample of 108 subjects, recruited within the first 5 days since admission at ICU. Our populational sample was higher than the expected sample size of 89 subjects for this analysis. In parallel, 16 patients presenting mild COVID-19 were enrolled, as a comparison group. To confirm COVID-19, as inclusion criteria, all mild and severe-COVID-19 subjects were positive for SARS-CoV-2 RNA detection. This study was approved by the Ethical Committee of HUCFF, number CAAE: 31240120.0.0000.5257, and all subjects (or representatives) signed the informed consent form.

For hospitalized subjects, severity evaluation by Sequential Organ Failure Assessment (SOFA) score (WHO Guidelines, 2020) was applied [18], and all patients were divided into two groups according to their outcomes: survivor and deceased. A total of 16 age-matched subjects presenting mild COVID-19, who did not require hospitalization, were recruited in Laboratory of Translational Neurosciences of the Federal University of the State of Rio de Janeiro (UNIRIO) during the same period of time.

For health control group, samples from 20 age-matched subjects were selected from the Laboratory of Translational Neurosciences of the Federal University of the State of Rio de Janeiro (UNIRIO) biorepository. To assure that the health group did not present previous SARS-CoV-2 infection, these samples were collected prior the COVID-19 pandemics.

For better understanding the potential of these biomarkers to predict death, all samples were collected as soon as possible since ICU admission (no longer than 5 days). Severe COVID-19 group was then divided between “deceased” and “alive” for statistical analysis. For severe COVID-19 patients the inclusion criteria were the need of ICU support, the consent to participate, and that blood samples could be collected within the first 5 days of ICU admission. Exclusion criteria were patients with more than 5 days in ICU, patients in whom the level of consciousness did not allow inquiring about their consent to participate in the research, those who did not consent, and those whose family could not be located or who did not consent. Mild COVID-19 subject inclusion criteria were the presence of flu-like symptoms and no need for hospitalization.

*Sample Collection*For all subjects, blood samples were collected in ethylenediamine tetraacetic acid (EDTA) and non-anticoagulation gel separator vacutainer tubes for obtainment of serum, plasma, and buffy coat. To separate these blood components, all samples were submitted to centrifugation at 1500–2000 × *g* for 10–15 min at room temperature. Plasma and serum samples were stored at − 20 °C in aliquots to avoid contamination. For long-term storage, all samples were kept at − 80 °C in the freezer 2 of LABNET, registered in a logbook with a registered location in the freezer.

Simultaneously, naso-oropharynx swab samples were also collected by sterilized swabs directly into Viral Transportation Medium (VTM) and then processed, at least, 30 min after collection. For some intubated subjects, swab samples were collected by bronchial aspirate.

### Data Collection

For further analysis, biochemical parameter levels, from blood tests performed at the hospital, reported comorbidities and clinical history of all severe subjects were collected from their medical records by the ICU responsible medical doctor for analysis.

### Neurological Biomarker Investigation

The Neuro 4-Plex Kit (Quanterix Corporation, Lexington, MA, USA) was used to perform plasma sample analysis of NfL, total TAU, GFAP, and UCH-L1 level quantification in blood. For that, the bead-based ultrasensitive technique was applied using SR-X instrument of SiMoA Platform (Quanterix Corporation, Lexington, MA, USA). The protocol was conducted according to manufacturer’s instructions. The assay includes calibration standard curves, for quantification, and analog and digital controls, for detection limit setting. Higher and lower controls and all samples were applied, in duplicate, according to the 1:4 dilution. Calibrators, controls, and samples were all applied in a 96-well plate, where paramagnetic carboxylated microspheres and detector buffer were added to the plate and it was incubated for 30 min at 35 °C at 800 rpm. After the incubation, the plate was washed, and substract (Streptavidin β-galactosidase (SβG)) was added and the plate was again incubated, now for 10 min at 35 °C and 800 rpm. After a second wash, it was incubated for 1 more minute under the same conditions, dried, and inserted into the SR-X equipment. The results, standard curves, and quantification are performed and recorded by Quanterix software and include dilution correction. These are the reference values for each biomarker: 0–500 pg/mL (NfL), 0–100 pg/mL (TAU), 0–1000 pg/mL (GFAP), and 0–10 ng/mL for (UCH-L1). Also, the lower detection limits for NfL, TAU, GFAP, and UCH-L1 are, respectively, 0.136 pg/mL, 0.0298 pg/mL, 0.276 pg/mL, and 4.03 pg/mL. The ranges for NfL, GFAP, UCH-L1, and TAU are 2000 pg/mL, 4000 pg/mL, 40,000 pg/mL, and 400 pg/mL, respectively. It is important to highlight that the plasma samples were diluted 1:4 for analysis and SiMoA software considers this for final concentration calculation.

### SARS-CoV-2 Detection and Quantification

All subjects were tested for SARS-CoV-2 RNA E region detection and quantification by Reverse Transcription–quantitative Polymerase Chain Reaction (RT-qPCR) assay (Bio-Manguinhos, Rio de Janeiro, Brazil). For that, viral RNA was purified by Janus 360 (PerkinElmer, Waltham, MA, USA) using magnetic bead automated technique. For quantification, an in-house ssRNA standard curve, previously validated, was applied. Samples presenting a CT > 35 were considered negative. This standard curve was obtained by purification, quantification, and serial-dilution of SARS-CoV-2 from cell culture [19].

### Statistical Analysis

The statistical analysis was performed using Prism 10.0.3 software (GraphPad Software). Each group was compared with its homologous biomarker from a one-way control group using the Kruskal–Wallis test followed by pos hoc Dunn’s test for data without Gaussian distribution. The non-parametric Mann–Whitney *U*-test and Student’s *t*-test were applied to determine whether the two groups alive and death were statistically different for categoric variables. For correlation, Spearman Correlation test was applied using IBM SPSS Statistics Software and R Studio. For multivariate analysis, Chi-square test and Factorial Analysis were used. For analysis, we considered 0 (zero) for Death and (1) for Survival, and for mortality association analysis, log-rank test was applied using SPSS software. For significance, *p* value was < 0.05 (two-tailed).

## Results

**Fig. 1:**
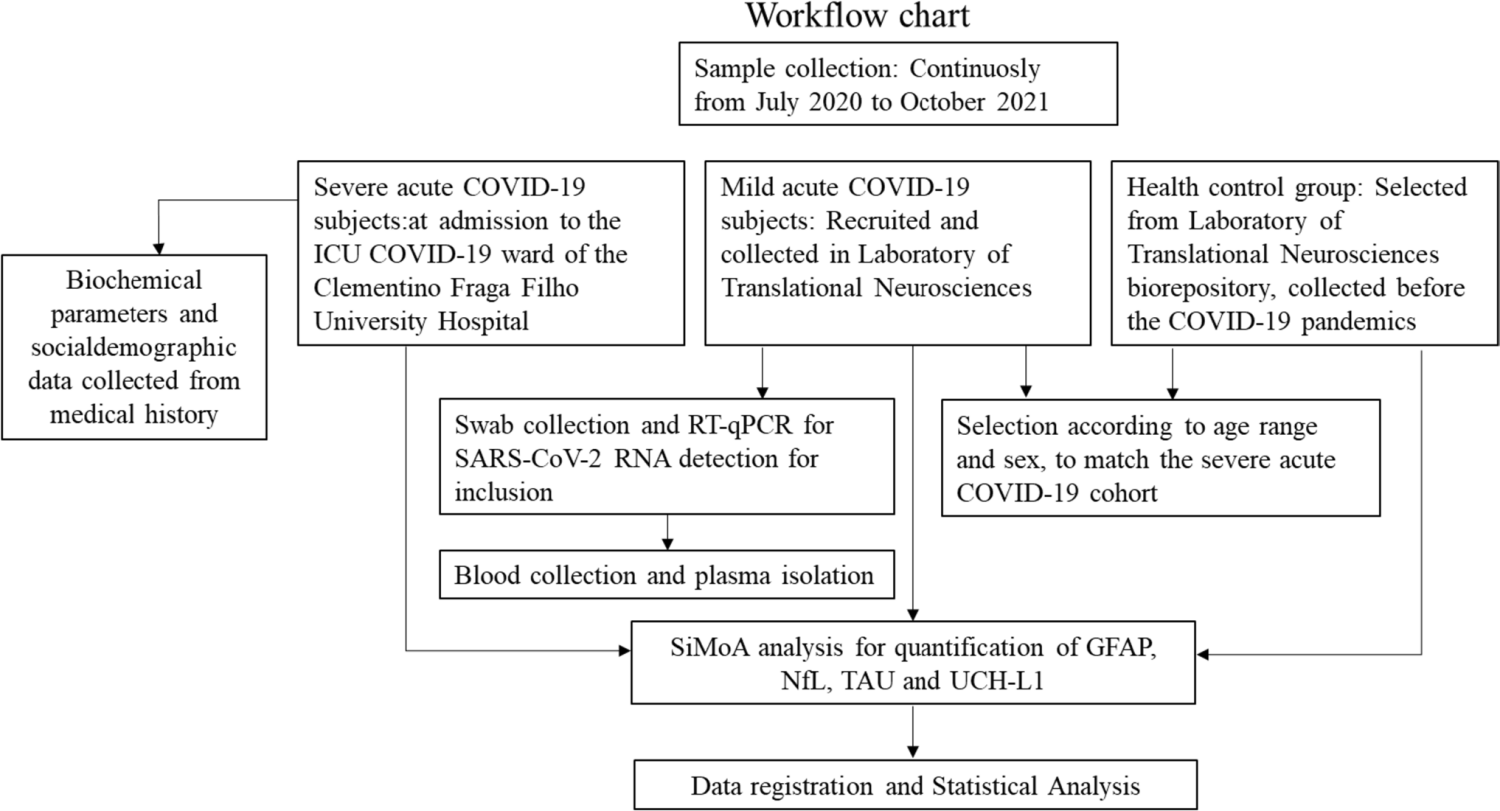
Workflow since subject selection and sample collection for all three groups included

Between July 2020 and October 2021, 1068 patients were attended at HUCFF/UFRJ with suspected COVID-19. From those, 89.7% (958) had the confirmed diagnosis by RT-qPCR or immunoassay for SARS-CoV-2 antigens detection, 9.17% (98) were diagnosed by clinical evaluation, and 1.12% (12) of the cases remained unsolved. The mean age for this group was 63.51 years old, median 65, and balanced between male (49.16%; 525) and female (50.84%; 543) individuals. A total of 706 patients were admitted in the ICU of the HUCFF/UFRJ. The workflow for sample collection and subjects’ selection is demonstrated in Fig. 1.

Our cohort included 108 subjects from this population. The mean age for this group was 62.92 years old (SD 14.14), median 66, and balanced between male (49.08%; 53) and female (50.92%; 55) individuals. Thirty-six 33.33% subjects presented any neurological manifestation and 37.96% (41) died (Table 1). For comparison, the gender (F/M) ratio for mild COVID-19 was 9/7 (56.25% and 43.75%) and for health control group was 8/12 (42.67% and 57.33%).

**Table 1:** Sociodemographical characteristics of severe COVID-19 cohort and comparative groups (Mild COVID-19 and Health Control)

Sociodemographic			
Severe COVID-19 cohort (*n* = 108)			
		Mean	SD
Age		62.92	** ± **16.17
Category		Frequency	Percentage
Gender	Female	55	50.92%
	Male	53	49.08%
Comorbidities	Present	108	100.00%
	Absent	0	0.00%
Type of comorbidities	Hypertension	83	76.85%
	Diabetes mellitus	36	33.33%
	Smoking	30	27.77%
Neurological manifestations	Present	36	33.33%
	Absent	72	66.67%
Outcome	Death	41	37.96%
	Survival	67	62.04%
Mild COVID-19 cohort (*n* = 16)			
		Mean	SD
Age		46.25	** ± **16.30
Category		Frequency	Percentage
Gender	Female	9	56.25%
	Male	7	43.75%
Comorbidities	Present	7	43.75%
	Absent	9	56.25%
Health Control Group (*n* = 20)			
		Mean	SD
Age		51.75	** ± **10.93
Category		Frequency	Percentage
Gender	Female	8	42.67%
	Male	12	57.33%

**Fig. 2:**
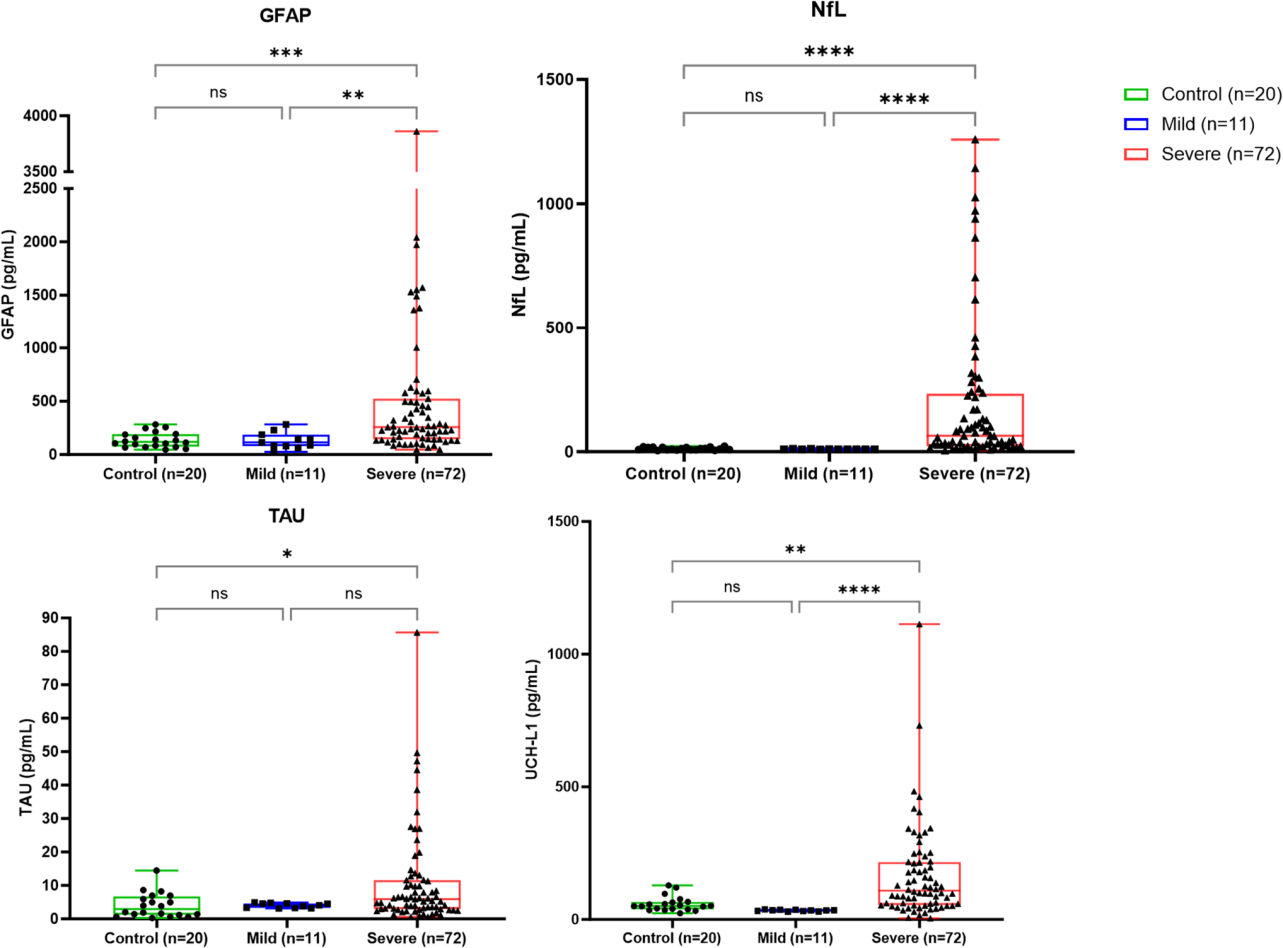
Biomarkers — **A** GFAP, **B** NfL, **C** TAU, and **D** UCH-L1 — levels in comparison of severe and mild COVID-19 groups and health controls. *p < 0.05, **p < 0.01, ***p < 0.001, ****p < 0.0001

To investigate the relationship among the candidate biomarkers and COVID-19 outcome, we underwent two different analyses including age-matching subjects from all groups (Table 2). First, we compared the plasma levels of NfL, GFAP, TAU, and UCH-L1 among severe and mild COVID-19 patients and health age-matched controls. We demonstrated significant higher levels of GFAP, NfL, TAU, and UCH-L1 (*p* ≤ 0.001) in severe group when compared to health controls. When severe COVID-19 group is compared to mild COVID-19, only TAU levels did not present significant difference. Neurological biomarker levels in individuals with mild COVID-19 did not show any difference when compared to a control group of healthy individuals (Fig. 2).

**Table 2:** Sociodemographical, biomarker, and biochemical parameter analysis between severe and mild COVID-19 subjects and health controls

		COVID-19 severity level		Severe COVID-19 patients’ outcome		*p*-value
	Health Control	Mild	Severe	Alive	Deceased	
Patients and health control	(*n* = 20)	(*n* = 11)	(*n* = 72)	(*n* = 44)	(*n* = 28)	
Age (mean)	51.75 ± 10.93	41.45 ± 15.02	62.61 ± 15.35	59.45 ± 14.79	67.84 ± 15.33	
SARS-Cov-2 E viral load (median)	NA	-	-	1332 ± 2794	10,600 ± 19,199	
Hospitalization (in days. media)	NA	NA	25.68 ± 46.98	19.5 ± 13.29	33.33 ± 69.00	
Hypertension	-	-	80.5%	79.54%	82.14%	
Diabetes	-	-	37.5%	34.09%	42.85%	
Neurologic disease	-	-	16.66%	11.36%	25%	
Pneumonia	-	-	78.12%	69.23%	92%	*p* = 0.0316
Hepatitis	-	-	19.44%	15.9%	25%	
Acute kidney injury	-	-	39.43%	18.60%	71.42%	*p* < 0.0001
Acute thrombotic event	-	-	9.85%	9.3%	10.71%	
Smoking	-	-	29.16%	31.81%	25%	
Oxygen supplementation	NA	NA	76%	62.8%	93.5%	*p* = 0.0052
SOFA index (median)	NA	NA	3.09 ± 2.78	2.5 ± 2.28	4 ± 3.17	*p* = 0.0153
Ferritin peak			6243 ± 15,230	1525 ± 3354	12,595 ± 21,465	*p* < 0.0001
D-dimer peak			7892 ± 10,971	5966 ± 10,352	9967 ± 11,261	*p* = 0.0081
TGO			43.29 ± 27.91	41.63 ± 30.17	45.37 ± 24.15	
TGP			32.71 ± 26.40	35.12 ± 29.19	28.60 ± 21.43	
GGT			124.6 ± 137.2	87.09 ± 74.79	174.9 ± 183.5	*p* = 0.0098
PCR peak			320.2 ± 910.9	143.4 ± 99.59	565.9 ± 1368	*p* < 0.0001
Urea			63.78 ± 47.74	63.05 ± 53.33	67.71 ± 41.45	
Creatinine			2.042 ± 2.701	2.284 ± 3.267	1.752 ± 1.539	
Lynphocite			1073 ± 799.5	1131 ± 811.6	1010 ± 783.0	
Monocyte			506.7 ± 363.8	485.8 ± 327.3	534.8 ± 408.9	
Neutrophiles			7483 ± 6490	7432 ± 7659	7460 ± 4326	
GFAP (pg/mL)	137.4 ± 70.98	130.5 ± 76.92	578.4 ± 852.0	362.4 ± 687.9	4201 ± 18,070	*p* = 0.0015
NfL (pg/mL)	12.49 ± 5.210	9.765 ± 0.9593	197.4 ± 292.8	109.9 ± 192.3	805.6 ± 2696	*p* = 0.0010
TAU (pg/mL)	4.027 ± 3.669	4.023 ± 0.6944	67.14 ± 485.2	9.059 ± 11.46	155.3 ± 748.8	*p* = 0.0401
UCH-L1 (pg/mL)	58.66 ± 28.09	34.18 ± 2.666	266.8 ± 886.6	108.5 ± 90.04	589.0 ± 1436	*p* < 0.0001

**Fig. 3:**
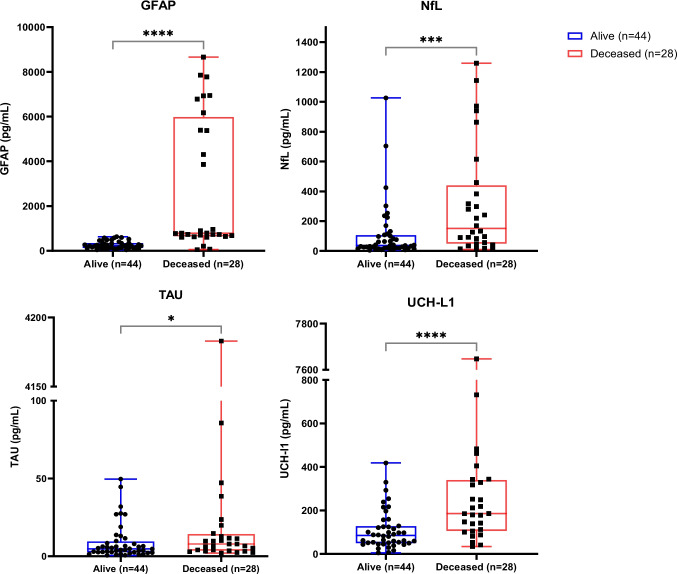
Biomarkers — **a** GFAP, **b** NfL, **c** TAU, and **d** UCH-L1 — levels in comparison between survivor and deceased subjects from the severe COVID-19 group. *p < 0.05, **p < 0.01, ***p < 0.001

These findings suggest that GFAP (*p* = 0.0015), NfL (*p* = 0.001), TAU (*p* = 0.0401), and UCH-L1 (*p* < 0.0001) plasmatic levels may be useful to predict poor COVID-19 outcome (Fig. 3).

**Fig. 4:**
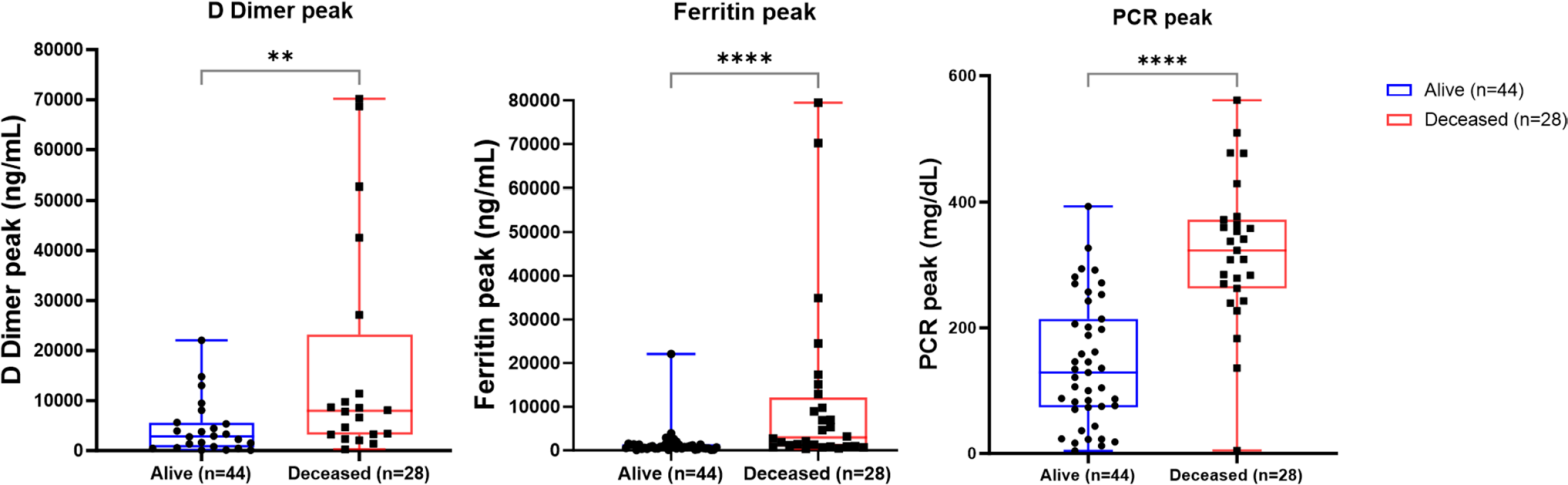
Peak serum D-dimer, ferritin, and PCR level comparison between survival (alive) and deceased severe COVID-19 patient

Analyzing our database, we could observe that Acute Kidney Injury (*p* < 0.0001), Pneumonia (*p* = 0.0316), Oxygen Supplementation (*p* = 0.0052), and SOFA index (*p* = 0.0153) in addition to the increased levels of biochemical biomarkers Serum Ferritin Peak (*p* < 0.0001), D-Dimer Peak (*p* < 0.05), Gamma-glutamyltransferase (GGT) (*p* < 0.05), and C-Reactive Protein (CRP) (*p* < 0.0001) were significantly related to death (Table 2; Fig. 4).

**Fig. 5:**
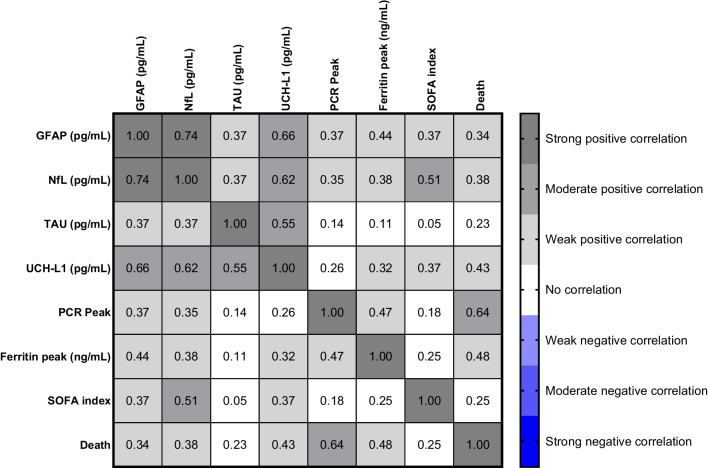
Spearman correlation graphic, considering the following parameters: GFAP, NfL, TAU, UCH-L1, PCR peak, ferritin peak, SOFA index, and death

In the Spearman Correlation analysis, the candidate biomarkers (GFAP, NfL, TAU, UCH-L1) and the Peak Serum Ferritin, D-Dimer Peak, GGT, and PCR levels were positively correlated with the SOFA index and death outcome, with the increase in these biomarkers being related to the negative result, except for the Peak Serum Ferritin level, which was not significant when compared with TAU levels, PCR peak, and SOFA index, and also the SOFA index was not significant when compared with the PCR peak (Table 3; Fig. 5).

**Table 3:** Multivariate analysis of biomarkers and outcomes (survivor and deceased) in severe COVID-19 subjects

Spearman’s rank correlation coefficient										
			GFAP	NfL	TAU	UCHL1	PCR peak	Ferritin peak	SOFA index	Death
Spearman’s rho	**GFAP**	Correlation coefficient	1.000	.749**	.375**	.673**	.393**	.464**	.389**	.354**
		Sig. (2-tailed)		.000	.001	.000	.001	.000	.001	.002
		*N*	72	72	72	72	72	72	72	72
	**NfL**	Correlation coefficient	.749**	1.000	.398**	.658**	.403**	.401**	.531**	.416**
		Sig. (2-tailed)	.000		.000	.000	.000	.000	.000	.000
		*N*	72	72	72	72	72	72	72	72
	**TAU**	Correlation coefficient	.375**	.398**	1.000	.566**	.192	.155	.092	.255*
		Sig. (2-tailed)	.001	.000		.000	.102	.190	.431	.028
		*N*	72	72	72	72	72	72	72	72
	**UCHL1**	Correlation coefficient	.673**	.658**	.566**	1.000	.323**	.348**	.397**	.465**
		Sig. (2-tailed)	.000	.000	.000		.005	.003	.000	.000
		*N*	72	72	72	72	72	72	72	72
	**PCR peak**	Correlation coefficient	.393**	.403**	.192	.323**	1.000	.489**	.225	.652**
		Sig. (2-tailed)	.001	.000	.102	.005		.000	.053	.000
		*N*	72	72	72	72	72	72	72	72
	**Ferritin peak**	Correlation coefficient	.464**	.401**	.155	.348**	.489**	1.000	.273*	.491**
		Sig. (2-tailed)	.000	.000	.190	.003	.000		.020	.000
		*N*	72	72	72	72	72	72	72	72
	**SOFA index**	Correlation coefficient	.389**	.531**	.092	.397**	.225	.273*	1.000	.281*
		Sig. (2-tailed)	.001	.000	.431	.000	.053	.020		.015
		*N*	72	72	72	72	72	72	72	72
	**Death**	Correlation coefficient	.354**	.416**	.255*	.465**	.652**	.491**	.281*	1.000
		Sig. (2-tailed)	.002	.000	.028	.000	.000	.000	.015	
		*N*	72	72	72	72	72	72	72	72

*Correlation is significant at the 0.05 level (2-tailed)

**Correlation is significant at the 0.01 level (2-tailed)

**Fig. 6:**
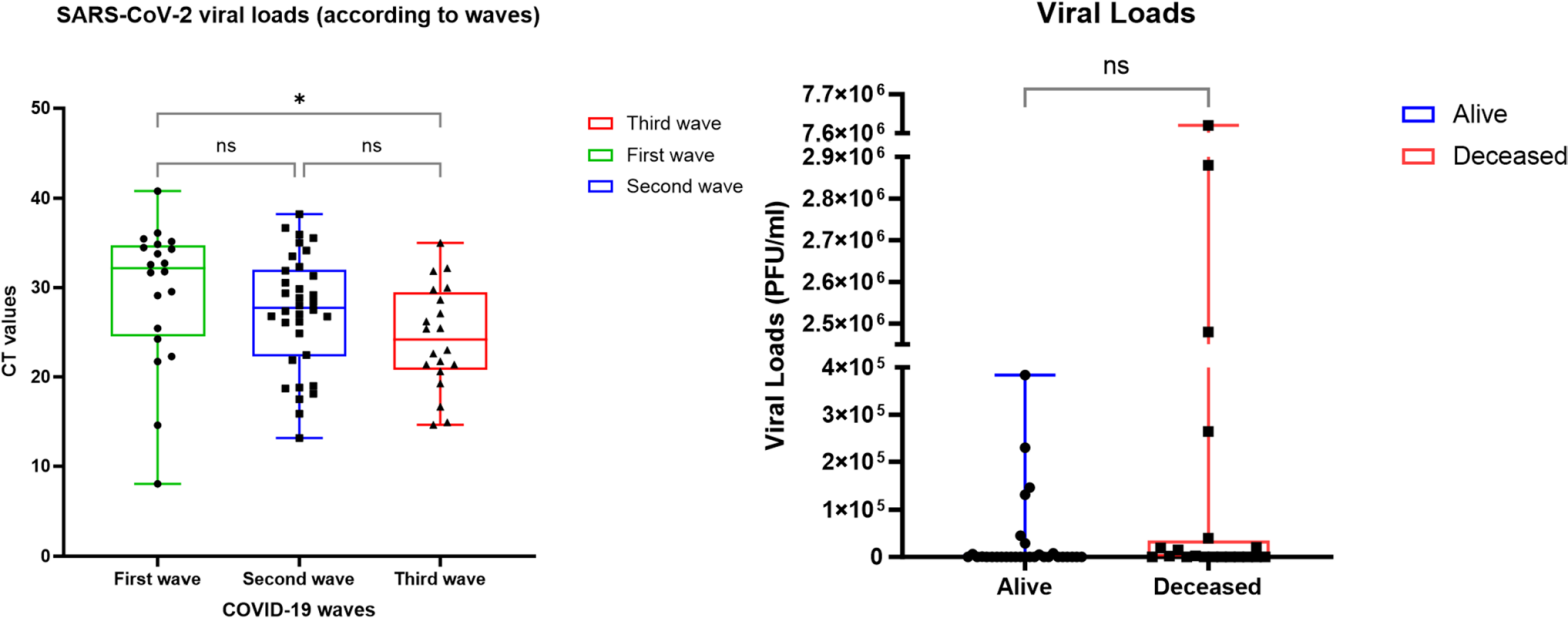
**a** Viral load (in CT — cycle threshold) comparison during time according to COVID-19 waves and variants in Brazil. **b** Viral load (PFU/mL) comparison between survival (alive) and deceased severe COVID-19 patients

Regarding SARS-CoV-2 viral load, we first considered the period of time of the sample collections and compared the SARS-CoV-2 viral loads presented by the subjects according to 1st, 2nd, and 3rd COVID-19 waves in Brazil, which were epidemiologically related to variants Beta, Gamma, and Delta, respectively, represented in CT values (cycle threshold), and we could observe a statistically significant decrease in CT values when third wave was compared to first wave (*p* < 0.05), meaning higher viral loads observed during third wave. Also, we ran other two evaluations, comparing viral loads between mild COVID-19 and severe COVID-19 groups and, within the severe COVID-19 group, between survivors and deceased. However, no statistically significant difference was observed for any of this group analysis (Fig. 6).

## Discussion

As far as we know, this is the first study to describe, simultaneously, the multisystemic and multiple biochemical parameters between survival and not survival severe COVID-19 patients associated to nervous system biomarkers NfL, GFAP, UCH-L1, and TAU. The relevance of this approach is supported by the capacity of SARS-CoV-2 to infect different tissues once it binds to Angiotensin-converting enzyme 2 (ACE2) receptor present in several organs [20]. There is strong evidence that SARS-CoV-2 infection evolves extrapulmonary systems, leading to multisystemic complications during and after the acute infection [21]. The early involvement of the nervous system is well documented and is associated with severe outcome [1, 13]. The neurological manifestations include encephalopathy, Guillain-Barré syndrome, acute disseminated encephalomyelitis, ischemic stroke, epilepsy, and neuropathy as part of a broad spectrum that continues to be described [13, 22–24]. The early implication of the nervous system, even without neurological clinical symptoms, can be identified by plasma biomarkers [25]. For a better understanding of how multisystemic COVID-19 is impacted by nervous system involvement and to validate candidate biomarkers for a practical approach of severity assessment, we enrolled 108 subjects from a 706-patient population admitted at the HUCFF ICU during the pandemics, between July 2020 and October 2021. For comparison, we included mild COVID-19 subjects that did not require hospitalization and only presented flu-like symptoms and a group of pre-pandemic health controls.

As NfL, GFAP, UCHL-1, and Tau protein are associated with senescence, the statistical analysis among severe COVID-19, mild COVID-19, and health control group biomarkers was matched by decade of life.

In our study, it was possible to observe that all investigated plasma biomarkers (GFAP, NfL, TAU, UCH-L1) presented significantly higher levels among severe COVID-19 when compared to health controls (*p* < 0.01). However, there was no statistical significance between the same biomarkers when mild COVID-19 and health control groups were compared. Among COVID-19-positive subjects (severe versus mild COVID-19), GFAP, NfL, and UCH-L1 presented statistical significance difference (*p* ≤ 0.01), but not total TAU. Our findings corroborate previous studies that have also investigated NfL and GFAP levels in severe and mild COVID-19 patients in comparison to health control individuals. NfL levels were significantly associated to severity and neurological manifestations of COVID-19 [8, 22, 26]. Kanberg et al. (2020) investigated and followed up 47 COVID-19 subjects and health controls for GFAP and NfL levels and observed that, among severe patients, GFAP levels decreased along time but NfL remained constant after 11.4 days [22]. Nonetheless, these authors not included UCH-L1 and TAU, which are potential biomarkers associated to neuron degeneration and did not include multisystemic involvement, other biochemical parameters, or outcome, as we did in the present study. Unfortunately, we could not follow up ICU subjects due to logistical resources, but we could also observe the relevant difference between ICU subjects versus mild COVID-19 and health control. Apparently, NfL levels tend to increase along time in severe/critical cases once it is related to neuronal injury and disease progression, while GFAP may indicate the astrocytic activation and injury as a first CNS response to infection [22]. The elevated results in GFAP described in our study are probably justified by sample collection in early stages of the disease. Similarly, Passos et al. (2022) suggest that GFAP is upregulated by SARS-CoV-2, demonstrating astrocyte disintegration and promoting the overexpression of inflammatory mediators related to neurological injury and degeneration [13].

In our study, we did not observe statistical significance in biomarker levels when mild COVID-19 patients and health controls were compared. Different from our findings, a previous analysis of 405 mild COVID-19 adolescents and young adult subjects showed significant difference between NfL and GFAP levels when these were compared to health controls [27]. Probably, for mild COVID-19, the age decades influenced the results.

Regarding TAU and UCH-L1, we found significant higher levels of these two biomarkers among severe COVID-19 patients when compared to mild COVID-19 subjects and health controls. Interestingly, even analyzing other outcomes (neurological involvement), significant elevations in TAU, NfL, and GFAP levels were also observed among hospitalized patients with and without neurological manifestations of SARS-CoV-2 infection [28]. These findings probably reinforce the early role of nervous system involvement as marker of disease severity [29], as we previously demonstrated [4, 10]. TAU levels have also been associated to critically illness polyneuropathy/myopathy [29], a relevant biomarker of severe axonal damage [30]. De Lorenzo et al. (2021) investigated 104 subjects and reported a significant difference in NfL and UCH-L1 levels between ICU and non-ICU COVID-19 patients [12]. Conversely, they did not find the same significance for GFAP and TAU levels, presumably due to different population, which included not only mild but also moderate patients in the non-ICU group.

Focusing on the severe/critical COVID-19 group, from 108 included subjects, 37.96% (41) had a death outcome and 28 out of 41 were included in the biomarker analyses. We found significant association among higher levels of NfL (*p* ≤ 0.001), GFAP (*p* < 0.0001), TAU (*p* < 0.05), and UCH-L1 (*p* < 0.0001) with death. This suggests the intense nervous system involvement, previously attributed by us and other authors, due to extended inflammatory response, axonal and neuronal injury, and astrocyte activation and damage [31]. GFAP and UCH-L1 are well-described biomarkers of traumatic brain injury, but when they are in higher levels concomitantly, they confer stronger evidence of severity in brain injury [15].

It is important to reinforce that, in our study, all investigated biomarkers were already reported significantly higher early in ICU admission for subjects with a further lethal outcome, as described by other authors [5, 12, 32–34]. As well as in the present study, other authors also found significant higher NfL levels in samples collected within the first 5 days in ICU, reinforcing this biomarker as early severity prognostic factor, and not a consequence of long-term hospitalization [32, 34].

Concomitantly, GFAP and UCH-L1 levels were found significantly higher among deceased patients, when compared to severe survival COVID-19 patients. Regarding GFAP, increased levels have been previously associated to critical and lethal outcomes in severe COVID-19 [12, 13, 28, 35]. Since astrocytes play an important role in brain response to viral infections, such as SARS-CoV-2, and inflammation, GFAP can be an important tool to predict astrocytic and CNS damage and neural injury [13, 35–38].

When it comes to UCH-L1, Tokic et al. (2022) investigated severity biomarkers and testosterone in severe COVID-19 male patients, then suggested that UCH-L1 may be related to neurological deficits in such population, reinforcing that this may be a relevant prognostic biomarker of neurological manifestation of COVID-19 [39].

Finally, corroborating our findings, TAU protein has been previously described closely related to death in severe COVID-19 [12, 28]. Apparently, SARS-CoV-2 infection may lead to hyperphosphorylation of TAU due to activation of both inflammatory signaling and oxidative stress pathways [40].

It is interesting that previous studies that investigated the same biomarkers presented slightly different results, reporting the significant difference in NfL, GFAP, and TAU levels between survivors and deceased severe COVID-19 patients, but they did not observe the same to UCH-L1 levels [12, 28].

By the inclusion of severe COVID-19 subjects, we collected sociodemographical, clinical data, and biochemical parameters from each patient’s medical history. So, we also analyzed biochemical biomarker levels from severe COVID-19 patients, and we observed that Peak Serum Ferritin (*p* < 0.0001), D-Dimer Peak (*p* < 0.05), GGT (*p* < 0.05), and PCR (*p* < 0.0001) levels were significantly higher among deceased subjects in comparison to survivors. Ferritin levels were previously associated to neuropathic pain after severe or longer COVID-19 and to death [8, 9]. D-Dimer Peak has been previously described as an important potential early biomarker in COVID-19, mainly when related to coagulation disorders and liver function [41, 42]; the same was observed for C-Reactive Protein [43–45], which was also early increased even before disease progression to severe COVID-19. GGT and D-Dimer have also been described, together increased early in severe COVID-19 cases, and other authors suggest the importance of considering them for decision-making right at admission to hospital once these results can be reached in a short period of time [46, 47]. When analyzed alone, both GGT and D-Dimer early increase was related to prolonged hospital stay, severity, and/or fatality [48, 49]. Frontera et al. (2021), however, did not observe the same correlation amid ferritin, D-dimer, GGT, PCR, and death and neither found significant association among ferritin and GFAP, NfL, TAU, and UCH-L1 [28].

Concomitantly, for deceased patients, we could also observe an important renal involvement in 71.42% of them, resulting in acute kidney injury (*p* < 0.0001), in agreement with previous authors that described the pathogenesis of COVID-19 and acute kidney injury related to death [50, 51]. Indeed, for the deceased subjects the same was observed for severe pulmonary involvement, once 92% of patients presented pneumonia (*p* = 0.0316) and 93.5% required oxygen support (*p* = 0.0052). These findings, together with biochemical parameters and neurological biomarkers, suggest secondary nervous system damage.

Unfortunately, our study presented some limitations due to the COVID-19 pandemic scenario and socioeconomical dynamics in Brazil. Because of that, we were not able to include more subjects or follow up COVID-19 patients during acute disease with multiple sample collections. In view of the pandemic situation in relation to COVID-19, our experience and previous searches allowed us to make preliminary analyses and make an important alert to the medical and scientific community about role of SARS-CoV-2 mechanism However, we are following up the severe COVID-19 survival group and mild COVID-19 group for long-COVID neurological manifestations [52]. Also, for the same reasons, we could not perform a multicentric study.

It is also important to add that we only investigated peripheral blood in order to suggest an alternative non-invasive relevant biomarker evaluation in a practical way using an ultrasensitive technique. The use of peripheral blood samples to investigate those biomarkers has been lately described by other authors [53], which investigated biomarkers and proinflammatory cytokines in serum and CSF for comparison. These previous researches validated peripheral blood sample collection for this purpose and even observed an increase in NfL levels in serum that was not observed in CSF samples.

When we look for the virus role in disease severity, viral load could be an important indicative of severity or tissue damage by viral replication. In general, in naso-oropharingeal samples, SARS-CoV-2 viral loads tend to increase reaching a peak by the symptom onset, decreasing until the viral clearance in 2 weeks. Meanwhile, they use to persist in stool, but the clinical significance of this is not clear yet [6, 7]. However, in our findings, we could not find a significant association of SARS-CoV-2 viral loads to COVID-19 severity and lethality, only when we compared virus dominant variants of concern during 1st, 2nd, and 3rd waves, which did not, in our study, directly interfere in subjects’ outcomes. Although some authors relate SARS-CoV-2 viral load to the extent of host inflammatory and immune responses and report the presence of SARS-CoV-2 in nervous system [54], both peripheral and central [29, 55], this direct relation is not clear. Jiao et al. (2021) suggest that this virus crosses the blood–brain barrier, infecting neurons as observed in non-human primates [56]. However, other studies involving animal models could not detect viral particles in brain tissue of adult Syrian hamsters [23]. In human patients, Edén et al. (2022) detected SARS-CoV-2 genomic RNA in plasma but not in CSF; N-antigen, an important replication biomarker, was also widely detected in plasma but not in CSF; and for S-antigen, it was rarely found in CSF [57]. Although in our study, patients were not examined for the assessment of viral load in the CSF, the levels of plasma biomarkers analyzed here are correlated and validated with the levels in the CSF. Therefore, these findings corroborate our results that higher viral loads seem not be related to severity outcome. For severe COVID-19 patients the inclusion criteria were the need of ICU support, the consent to participate, and that blood samples could be collected within the first 5 days of ICU admission. Exclusion criteria were patients with more than 5 days in ICU, patients in whom the level of consciousness did not allow inquiring about their consent to participate in the research, those who did not consent, and those whose family could not be located or who did not consent.

This is the first Brazilian study considering mild and severe COVID-19 patients investigated simultaneously by four biomarkers (NfL, GFAP, TAU, and UCH-L1), biochemical parameters, viral load, comorbidities, and outcome analysis. This approach allows us a better understanding about the multisystemic COVID-19 conditions and identify predictive risk factors of survival or death associated to nervous system involvement biomarkers, even in patients without neurological clinic manifestation. Different of previously reported, we could observe a statistical significance for all these four biomarkers, in peripheral blood (non-invasive collect sample), as potential predictors of poor outcome in severe COVID-19 significantly associated with SOFA index, acute kidney injury, pneumonia, and need of supplementary oxygen support. In this context, our results contribute demonstrating important risk factors to be considered in decision-making for each specific case.

Thus, we can conclude that NFL, GFAP, UCH-L1, and TAU are early biomarkers of the severity of SARS-COV-2 infection and may play an important role in therapeutic decision-making in the acute phase of the disease. Also, early in severe COVID-19 with poor outcome, Peak Serum Ferritin, D-Dimer, GGT, and PCR are already elevated and viral loads do not seem to be related to SARS-CoV-2 infection severity and lethality.

### Supplementary Information

Below is the link to the electronic supplementary material.

Supplementary file1 (DOCX 345 KB)

## Data Availability

The datasets generated during and/or analyzed during the current study are not publicly available because these are original data, not shown or published previously. Datasets are saved in the Laboratory of Translational Neurosciences cloud server and are available from the corresponding authors on reasonable request.

## References

[1] Forchette L, Sebastian W, Liu T (2021). A comprehensive review of COVID-19 virology, vaccines, variants, and therapeutics. Curr Med Sci.

[2] Zhang Y, Zhang H, Zhang W (2022). SARS-CoV-2 variants, immune escape, and countermeasures. Front Med.

[3] World Health Organization WHO [internet] (2023) Switzerland. World Health Organization. Statement on the fifteenth meeting of the IHR (2005) Emergency Committee on the COVID-19 pandemic. available from: https://www.who.int/news/item/05-05-2023-statement-on-the-fifteenth-meeting-of-the-international-health-regulations-(2005)-emergency-committee-regarding-the-coronavirus-disease-(covid-19)-pandemic. Accessed 28 June 2023

[4] de Cavalcante LTF, da Fonseca GC, Amado Leon LA, Salvio AL, Brustolini OJ, Gerber AL (2022). Buffy coat transcriptomic analysis reveals alterations in host cell protein synthesis and cell cycle in severe COVID-19 patients. Int J Mol Sci.

[5] Petrella C, Zingaropoli MA, Ceci FM, Pasculli P, Latronico T, Liuzzi GM (2023). COVID-19 affects serum brain-derived neurotrophic factor and neurofilament light chain in aged men: implications for morbidity and mortality. Cells.

[6] Walsh KA, Jordan K, Clyne B, Rohde D, Drummond L, Byrne P (2020). SARS-CoV-2 detection, viral load and infectivity over the course of an infection. J Infect.

[7] Zheng S, Fan J, Yu F, Feng B, Lou B, Zou Q (2020). Viral load dynamics and disease severity in patients infected with SARS-CoV-2 in Zhejiang Province, China, January–March 2020: retrospective cohort study. BMJ.

[8] Magdy R, Eid RA, Fathy W, Abdel-Aziz MM, Ibrahim RE, Yehia A (2022). Characteristics and risk factors of persistent neuropathic pain in recovered COVID-19 patients. Pain Med.

[9] Martins-Filho PR, Tavares CSS, Santos VS (2020). Factors associated with mortality in patients with COVID-19. A quantitative evidence synthesis of clinical and laboratory data. Eur J Intern Med.

[10] Amado LA, da Coelho WLCNP, Alves ADR, de Carneiro VCS, da Moreira OC, de Paula VS (2023). Clinical profile and risk factors for severe COVID-19 in hospitalized patients from Rio de Janeiro, Brazil: comparison between the first and second pandemic waves. J Clin Med.

[11] Tenforde MW, Kim SS, Lindsell CJ, Billig Rose E, Shapiro NI, Files DC (2020). Symptom duration and risk factors for delayed return to usual health among outpatients with COVID-19 in a multistate health care systems network — United States, March–June 2020. MMWR Morb Mortal Wkly Rep.

[12] De Lorenzo R, Loré NI, Finardi A, Mandelli A, Cirillo DM, Tresoldi C (2021). Blood neurofilament light chain and total tau levels at admission predict death in COVID-19 patients. J Neurol.

[13] Passos FRS, Heimfarth L, Monteiro BS, Corrêa CB, de Moura TR, de Araújo AAS (2022). Oxidative stress and inflammatory markers in patients with COVID-19: potential role of RAGE, HMGB1, GFAP and COX-2 in disease severity. Int Immunopharmacol.

[14] Gaetani L, Blennow K, Calabresi P, Di Filippo M, Parnetti L, Zetterberg H (2019). Neurofilament light chain as a biomarker in neurological disorders. J Neurol Neurosurg Psychiatry.

[15] Wang KKW, Kobeissy FH, Shakkour Z, Tyndall JA (2021). Thorough overview of ubiquitin C-terminal hydrolase-L1 and glial fibrillary acidic protein as tandem biomarkers recently cleared by US Food and Drug Administration for the evaluation of intracranial injuries among patients with traumatic brain injury. Acute Med Surg.

[16] Ashton NJ, Hye A, Rajkumar AP, Leuzy A, Snowden S, Suárez-Calvet M (2020). An update on blood-based biomarkers for non-Alzheimer neurodegenerative disorders. Nat Rev Neurol.

[17] Hamano T, Enomoto S, Shirafuji N, Ikawa M, Yamamura O, Yen S-H (2021). Autophagy and Tau protein. Int J Mol Sci.

[18] World Health Organization WHO [internet] (2021) Switzerland. World Health Organization. Living guidance for clinical management of COVID-19. available from:) https://www.who.int/publications-detail-redirect/WHO-2019-nCoV-clinical-2021-2. Accessed 28 June 2023

[19] Mares-Guia MAMM, Paiva AAP, Mello VM, Eller CM, Salvio AL, Nascimento FF (2022). Effectiveness of household disinfection techniques to remove SARS-CoV-2 from cloth masks. Pathogens.

[20] Beyerstedt S, Casaro EB, Rangel ÉB (2021). COVID-19: angiotensin-converting enzyme 2 (ACE2) expression and tissue susceptibility to SARS-CoV-2 infection. Eur J Clin Microbiol Infect Dis.

[21] Zheng KI, Feng G, Liu W, Targher G, Byrne CD, Zheng M (2021). Extrapulmonary complications of COVID-19: a multisystem disease?. J Med Virol.

[22] Kanberg N, Ashton NJ, Andersson L-M, Yilmaz A, Lindh M, Nilsson S (2020). Neurochemical evidence of astrocytic and neuronal injury commonly found in COVID-19. Neurology.

[23] Imai M, Iwatsuki-Horimoto K, Hatta M, Loeber S, Halfmann PJ, Nakajima N (2020). Syrian hamsters as a small animal model for SARS-CoV-2 infection and countermeasure development. Proc Natl Acad Sci U S A.

[24] Helms J, Kremer S, Merdji H, Clere-Jehl R, Schenck M, Kummerlen C (2020). Neurologic features in severe SARS-CoV-2 infection. N Engl J Med.

[25] Khalil M, Teunissen CE, Otto M, Piehl F, Sormani MP, Gattringer T (2018). Neurofilaments as biomarkers in neurological disorders. Nat Rev Neurol.

[26] Paterson RW, Benjamin LA, Mehta PR, Brown RL, Athauda D, Ashton NJ (2021). Serum and cerebrospinal fluid biomarker profiles in acute SARS-CoV-2-associated neurological syndromes. Brain Commun.

[27] Havdal LB, Berven LL, Selvakumar J, Stiansen-Sonerud T, Leegaard TM, Tjade T (2022). Neurological involvement in COVID-19 among non-hospitalized adolescents and young adults. Front Neurol.

[28] Frontera JA, Boutajangout A, Masurkar AV, Betensky RA, Ge Y, Vedvyas A (2022). Comparison of serum neurodegenerative biomarkers among hospitalized COVID-19 patients versus non-COVID subjects with normal cognition, mild cognitive impairment, or Alzheimer’s dementia. Alzheimers Dement.

[29] Frithiof R, Rostami E, Kumlien E, Virhammar J, Fällmar D, Hultström M (2021). Critical illness polyneuropathy, myopathy and neuronal biomarkers in COVID-19 patients: a prospective study. Clin Neurophysiol.

[30] Caprelli MT, Mothe AJ, Tator CH (2018). Hyperphosphorylated tau as a novel biomarker for traumatic axonal injury in the spinal cord. J Neurotrauma.

[31] Fontes-Dantas FL, Fernandes GG, Gutman EG, De Lima EV, Antonio LS, Hammerle MB (2023). SARS-CoV-2 Spike protein induces TLR4-mediated long-term cognitive dysfunction recapitulating post-COVID-19 syndrome in mice. Cell Rep.

[32] Erben Y, Prudencio M, Marquez CP, Jansen-West KR, Heckman MG, White LJ (2022). Neurofilament light chain and vaccination status associate with clinical outcomes in severe COVID-19. iScience.

[33] Masvekar RR, Kosa P, Jin K, Dobbs K, Stack MA, Castagnoli R et al (2022) Prognostic value of serum/plasma neurofilament light chain for COVID-19 associated mortality. Ann Clin Transl Neurol 9(5):622-632. 10.1002/acn3.51542 10.1002/acn3.51542 PMC9082006 35313387

[34] Prudencio M, Erben Y, Marquez CP, Jansen-West KR, Franco-Mesa C, Heckman MG (2021). Serum neurofilament light protein correlates with unfavorable clinical outcomes in hospitalized patients with COVID-19. Sci Transl Med.

[35] Fällmar D, Rostami E, Kumlien E, Ashton NJ, Jackmann S, Pavel R (2022). The extent of neuroradiological findings in COVID-19 shows correlation with blood biomarkers, Glasgow coma scale score and days in intensive care. J Neuroradiol.

[36] Boldrini M, Canoll PD, Klein RS (2021). How COVID-19 affects the brain. JAMA Psychiat.

[37] McMahon CL, Staples H, Gazi M, Carrion R, Hsieh J (2021). SARS-CoV-2 targets glial cells in human cortical organoids. Stem Cell Rep.

[38] Lavi E, Cong L (2020). Type I astrocytes and microglia induce a cytokine response in an encephalitic murine coronavirus infection. Exp Mol Pathol.

[39] Tokic D, Mikacic M, Kumric M, Ticinovic Kurir T, Rancic I, Martinovic D (2022). Association between brain injury markers and testosterone in critically-ill COVID-19 male patients. Microorganisms.

[40] Reiken S, Sittenfeld L, Dridi H, Liu Y, Liu X, Marks AR (2022). Alzheimer’s-like signaling in brains of COVID-19 patients. Alzheimers Dement.

[41] Azkur AK, Akdis M, Azkur D, Sokolowska M, van de Veen W, Brüggen M-C (2020). Immune response to SARS-CoV-2 and mechanisms of immunopathological changes in COVID-19. Allergy.

[42] Baroiu L, Lese AC, Stefanopol IA, Iancu A, Dumitru C, Ciubara AB (2022). The role of D-dimers in the initial evaluation of COVID-19. Ther Clin Risk Manag.

[43] Smilowitz NR, Kunichoff D, Garshick M, Shah B, Pillinger M, Hochman JS (2021). C-reactive protein and clinical outcomes in patients with COVID-19. Eur Heart J.

[44] Sadeghi-Haddad-Zavareh M, Bayani M, Shokri M, Ebrahimpour S, Babazadeh A, Mehraeen R (2021). C-reactive protein as a prognostic indicator in COVID-19 patients. Interdiscip Perspect Infect Dis.

[45] Ali N (2020). Elevated level of C-reactive protein may be an early marker to predict risk for severity of COVID-19. J Med Virol.

[46] Kumar-M P, Mishra S, Jha DK, Shukla J, Choudhury A, Mohindra R (2020). Coronavirus disease (COVID-19) and the liver: a comprehensive systematic review and meta-analysis. Hepatol Int.

[47] Kasapoglu B, Yozgat A, Tanoglu A, Can G, Sakin YS, Kekilli M (2021). Gamma-glutamyl-transferase may predict COVID-19 outcomes in hospitalised patients. Int J Clin Pract.

[48] Liu J, Yu C, Yang Q, Yuan X, Yang F, Li P (2021). The clinical implication of gamma-glutamyl transpeptidase in COVID-19. Liver Res.

[49] Nemec HM, Ferenczy A, Christie BD, Ashley DW, Montgomery A (2022). Correlation of D-dimer and outcomes in COVID-19 patients. Am Surg.

[50] Ng JH, Hirsch JS, Hazzan A, Wanchoo R, Shah HH, Malieckal DA (2021). Outcomes among patients hospitalized with COVID-19 and acute kidney injury. Am J Kidney Dis.

[51] Hilton J, Boyer N, Nadim MK, Forni LG, Kellum JA (2022). COVID-19 and acute kidney injury. Crit Care Clin.

[52] Gutman E, Salvio A, Fernandes R, Duarte L, Raposo-Vedovi J, Alcaraz H (2023). Long COVID: plasma levels of neurofilament light chain in mild COVID-19 patients with neurocognitive symptoms. In Rev.

[53] Guasp M, Muñoz-Sánchez G, Martínez-Hernández E, Santana D, Carbayo Á, Naranjo L (2022). CSF biomarkers in COVID-19 associated encephalopathy and encephalitis predict long-term outcome. Front Immunol.

[54] Shivshankar P, Karmouty-Quintana H, Mills T, Doursout M-F, Wang Y, Czopik AK (2022). SARS-CoV-2 infection: host response, immunity, and therapeutic targets. Inflammation.

[55] DeKosky ST, Kochanek PM, Valadka AB, Clark RSB, Chou SH-Y, Au AK (2021). Blood biomarkers for detection of brain injury in COVID-19 patients. J Neurotrauma.

[56] Jiao L, Yang Y, Yu W, Zhao Y, Long H, Gao J (2021). The olfactory route is a potential way for SARS-CoV-2 to invade the central nervous system of rhesus monkeys. Signal Transduct Target Ther.

[57] Edén A, Grahn A, Bremell D, Aghvanyan A, Bathala P, Fuchs D (2022). Viral antigen and inflammatory biomarkers in cerebrospinal fluid in patients with COVID-19 infection and neurologic symptoms compared with control participants without infection or neurologic symptoms. JAMA Netw Open.

