# Supplementary material for: High Levels of NfL, GFAP, TAU, and UCH-L1 as Potential Predictor Biomarkers of Severity and Lethality in Acute COVID-19
Source: Mol Neurobiol. 2023 Nov 24;61(6):3545–58. doi: 10.1007/s12035-023-03803-z (PMC11087339; doi:10.1007/s12035-023-03803-z)
Supplement: Supplementary file 1 — Supplementary file1 (DOCX 345 KB) [file 12035_2023_3803_MOESM1_ESM.docx]

**Supplementary material**


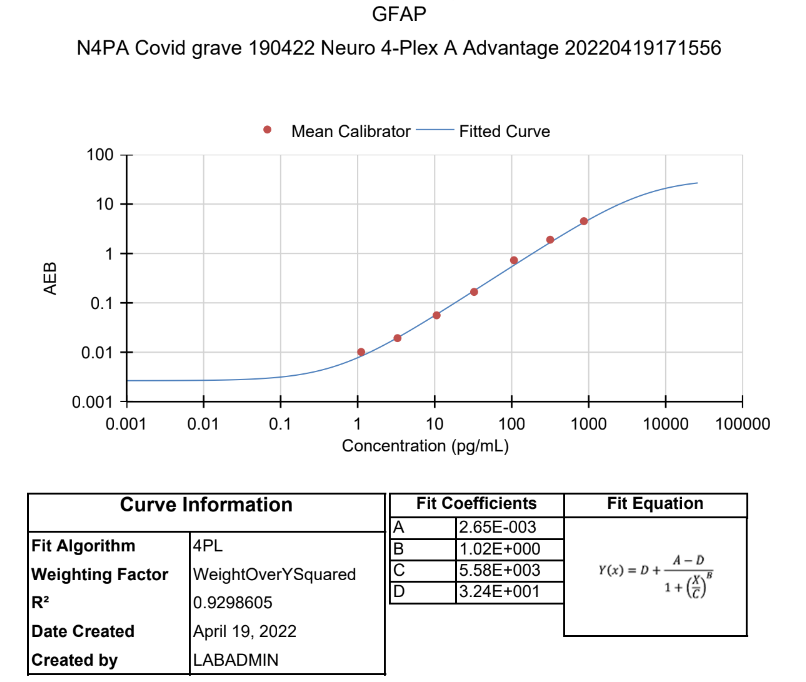
(a)


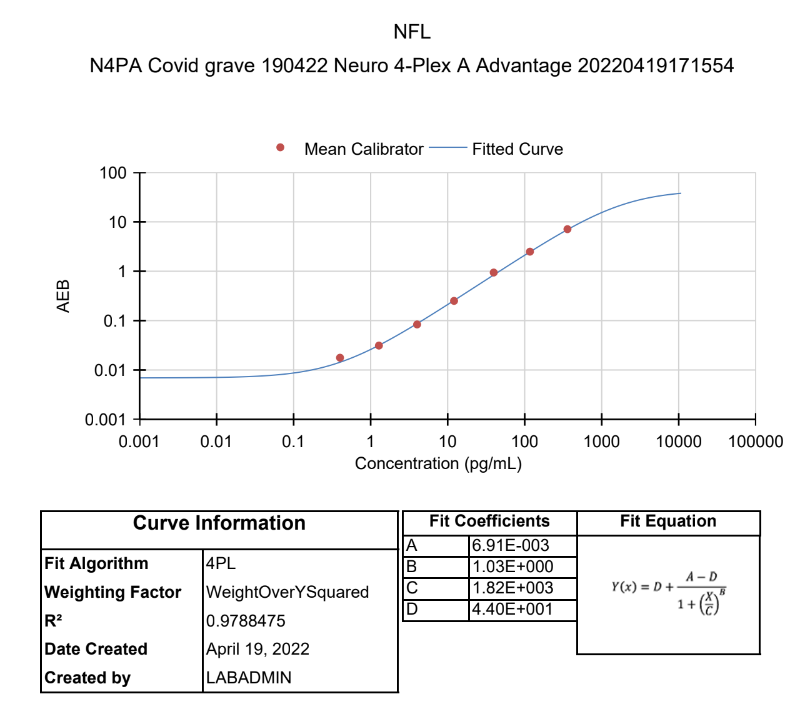


(b)


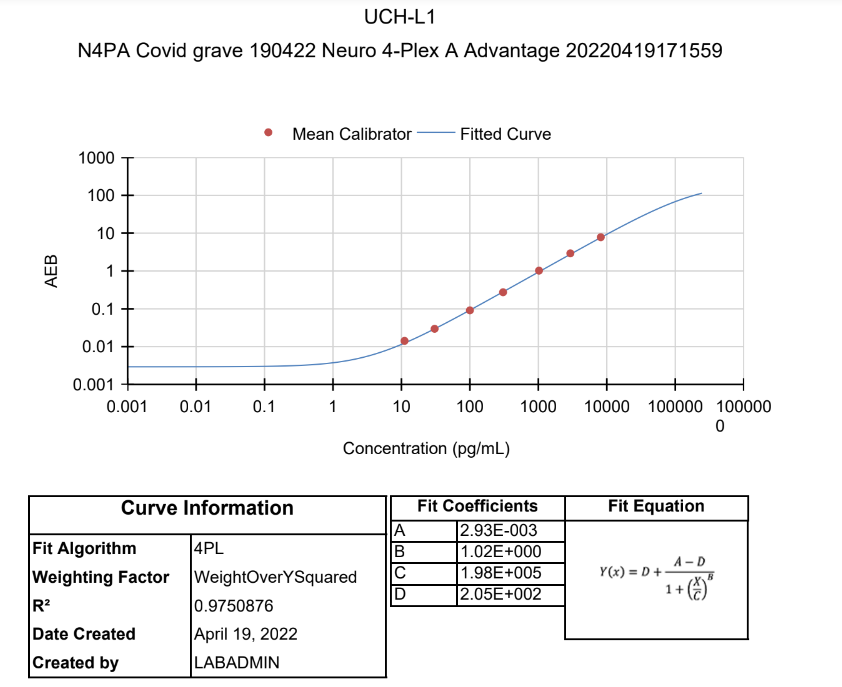
(c)


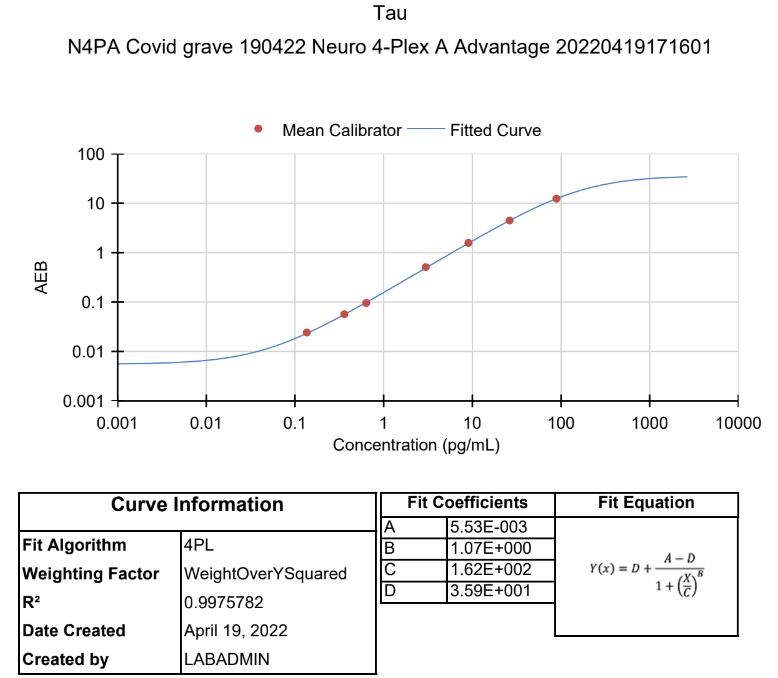


(d)

Fig S1: Calibration curves of biomarkers (a) GFAP, (b) NfL, (c) TAU and (d) UCH-L1 for the SiMoA assays validation.
